# Analysis of the stability and affinity of BlaR-CTD protein to β-lactam antibiotics based on docking and mutagenesis studies

**DOI:** 10.1186/s13036-019-0157-4

**Published:** 2019-03-29

**Authors:** Jianan Ning, Saeed Ahmed, Guyue Cheng, Ting Chen, Yulian Wang, Dapeng Peng, Zonghui Yuan

**Affiliations:** 1 National Reference Laboratory of Veterinary Drug Residues (HZAU) and MOA Key Laboratory for the Detection of Veterinary Drug Residues in Foods, Huazhong Agricultural University, Wuhan, 430070 China; 2 MOA Laboratory for Risk Assessment of Quality and Safety of Livestock and Poultry Products, Huazhong Agricultural University, Wuhan, 430070 China

**Keywords:** β-Lactam antibiotics, BlaR-CTD, Docking, Site-directed mutagenesis, Stability, Affinity

## Abstract

Owing to the thermal instability and low affinity of BlaR-CTD to some β-lactams, the receptor assay based on BlaR-CTD is limited in the detection of abundant variety of drugs and the result is often unstable. In this study, the three-dimensional structure of BlaR-CTD from *Bacillus licheniformis* ATCC14580 was constructed by homologous modeling based on the crystal structure of BlaR-CTD from *B. licheniformis* 749/I, and the binding sites of this protein to 40 β-lactams were also obtained by molecular docking. To improve the stability and affinity of the protein, 23 mutant proteins were designed based on docking and homologous alignment results as well as by inserting disulfide bond and building the salt bridge. The mutation was rationality evaluated by SIFT and PloyPhen2 software. The heterologous expressed and purified mutant proteins were then subjected to the activity and stability assay. It was shown that among all mutant proteins, I188K/S19C/G24C, A138E/R50C/Q147C and S190Y/E183C/I188K respectively exhibited a higher affinity to 33, 22 and 21 β-lactams than the wild-type protein, while I188K/S19C/G24C exhibited the best stability. This may due to that the conformation of the active site in mutant protein I188K/S19C/G24C changed, and the random coli in the surface of protein activity increased. Our study suggests a possible structure-function relationship on the stability and affinity of BlaR-CTD, which provides new insights into protein rational design study and lays a solid foundation for establishing the receptor-based screening assay for the detection of β-lactam residues.

**Electronic supplementary material:**

The online version of this article (10.1186/s13036-019-0157-4) contains supplementary material, which is available to authorized users.

## Introduction

The penicillin binding protein (PBP) BlaR is a signal transduction membrane protein which induces the synthesis of β-lactamase. The C-terminal domain of BlaR (BlaR-CTD) protein is located in the extracellular region which acts as a drug binding site [19]. BlaR-CTD, a penicillin-binding protein. BlaR-CTD is firstly the sensor domain of a penicillin receptor that is acylated by penicillin. This protein can identify and bind to a variety of β-lactams [8, 12]. In the active site of the protein, STYK, serine was a key amino acid (AA) that can participate in the binding of β-lactams [31].

BlaR-CTD protein has been used to detect the β-lactam residues in the receptor-based screening assay, such as a biological fluid method using colloidal gold labeled receptor protein [7] and an receptor-based enzyme linked immune-sorbent assay developed by our lab in which BlaR-CTD from *B. licheniformis* ATCC14580 was immobilized on the plate [25]. The limits of detection for 11 β-lactams were lower than the maximum residue limits regulated by European Union. However, the study showed that BlaR-CTD protein had a poor affinity for some β-lactams, e.g. cephalexin and cefadroxil. In addition, the thermal stability of the protein was poor that the protein activity only remained 70% at − 20 °C after 1 month, and lost fast at 4 °C [25].

Presently, site-directed mutagenesis was an effective means for the structural modification of protein [14], such as, improving the enzymatic activity and stability [9, 15]. Contreras-Martel et al. obtained a wild-type and the mutant PBP2b from *Streptococcus pneumoniae* by heterologous expression in *E. coli*, and the structure analysis showed that the mutation of AAs around the active site could affect the distribution of charge and polarity, preventing the entry of substrate into the active site, thus results in a bacterial resistance against β-lactam drugs [4]. Powell et al. mutated the AAs around the active site of the PBP2 from *Neisseria gonorrhoeae*, and the results showed that the acylation efficiency for penicillin G was reduced [26]. Therefore, the mutation site should avoid the active site and active site of the protein when the structure of BlaR-CTD protein was reconstructed. Molecular docking can quickly simulates the binding mode between a protein and a ligand, and analyze the key AAs involved in the binding, such as, AAs forming a hydrogen bond(s) with the drug [33]. Moreover, disulfide bond played an important role in the structural stability of proteins. It was demonstrated that the protein expression and enzyme activity of β-mannanase from *Aspergillus niger* BK01 (ManBK) were reduced when the conserved C171-C174 disulfide bond was missing, indicating that disulfide bond has a crucial in the stability of [15]. Through the site-directed mutagenesis of PBP3 from *Streptococcus pneumoniae* R6, the thermal stability of the mutant A353C/E393C was increased while the affinity of the mutant did not change [32].

In this study, 23 mutant proteins of BlaR-CTD from *Bacillus licheniformis* ATCC14580 were designed by homologous modeling, molecular docking, and prediction of mutation site in software, disulfide bond inserting and salt bridge building. The mutant proteins were obtained by site-directed mutagenesis and heterologous expression in *E. coli*. Based on the results of activity and stability tests, I188K/S19C/G24C showed equal or higher affinity to 33 β-lactams and better stability as compared to the wild-type protein. This study for the first time modified BlaR-CTD protein by rational design and site-directed mutagenesis, and the mutant protein I188K/S19C/G24C exhibited not only the improvement in stability but also higher affinity than the wild-type. Current study offers a powerful base for establishing the screening method for the detection of β-lactam residues.

## Materials and methods

### Chemicals

Penicillin G, nafcillin, dicloxacillin, ceftiofur, cefquinome, cefuroxim, carbenicillin and flucloxacillin were bought from Dr. Ehrenstorfer (Deisenhofen, Germany). Ampicillin, amoxicillin, oxacillin, azlocillin, penicillin V, cloxacillin, cefalotin, cefoperazone, cefazolin, cefalexin, ceftriaxone, cefadroxil, cefepime, cefradin, latamoxef, cefixim, ceftazidim, cefoxitin, sulbenicillin, ticarcillin and aztreonam were supplied by the National Institute for the Control of Pharmaceutical and Biological Products (Beijing, China). Piperacillin and cefalonium were from Toronto Research Chemicals (Toronto, Canada). Cefapirin was from Witega (Berlin, Germany). Benzylpenicillin was from European Directorate for the Quality of Medicines & HealthCare (Strasbourg, France). Kanamycin was obtained from TianYuan (Wuhan, China). 1-ethyl-3-(3-dimethylaminopropyl) carbodiimide hydrochloride (EDC) and *N*-hydroxysuccinimide (NHS) were purchased from Sigma-Aldrich (St. Louis, MO, USA). Bovine serum albumin (BSA) was obtained from Bovogen (East Keilor, VIC, Australia). β-isopropyl-d-thiogalactopyranoside (IPTG) and horseradish peroxidase (HRP) were purchased from Solarbio (Beijing, China). 3,3′,5,5′-tetramethylbenzidine (TMB) and Ni^2+^ Sepharose Fastflow were from Thermo Fisher Scientific (Waltham, MA, USA). Luria-Bertani (LB) broth and agar were purchased form Qingdao Hope Bio-Technology (Qingdao, China). All other reagents were purchased from Sinopharm Chemical Reagent Co., Ltd. (Shanghai, China) and were all of analytical grade.

### Bacteria and plasmid

*E. coli* BL21 (DE3) and *E*. *coli* DH5α were purchased from TransGen Biotech (Wuhan, China). Recombinant plasmid pET-28a(+)-BlaR-CTD was obtained from the National Reference Laboratory of Veterinary Drug Residues (HZAU), Wuhan, China [25].

### Homologous modeling and molecular docking

By sequence alignment using NCBI-BLAST (Basic Local Alignment Search Tool), the BlaR-CTD protein from *B. licheniformis* 749/I was chosen as a template to build the three dimensional (3D) structure model of BlaR-CTD protein from *B. licheniformis* ATCC14580 (the AA sequence identity is 91%). Using homologous modelling motif of Sybyl-X2.0 software (Tripos, USA), the 3D structure of BlaR-CTD protein was obtained based on the crystal structure of BlaR-CTD protein from *B. licheniformis* 749/I (PDB ID: 1NRF) [16]. Sybyl-X2.0 was also used for molecular docking between BlaR-CTD protein and 40 β-lactams, and the key AAs involved in the formation of hydrogen bonds between the protein and each drug were obtained. The AAs within 5 Å of the active pocket were considered as AAs that may be associated with affinity.

The 3D structure of mutant protein was also obtained by homologous modelling and the key AAs involved in the formation of hydrogen bonds between mutant protein and each β-lactam were obtained by molecular docking using Sybyl-X2.0 software, as described above. Structural model was visualized by PYMOL (www.pymol.org).

### Design of mutational sites

SIFT (https://sift.bii.a-star.edu.sg) was used to predict whether an AA substitution effects on protein function based on the degree of conservation of AA residues in a sequence alignments derived from closely related sequences [21]. PloyPhen2 (http://genetics.bwh.harvard.edu/pph2/) was used to predict the possible impact of an AA substitution on the structure and function of a protein based on a number of features comprising the sequence, phylogenetic and structural information characterizing the substitution [1]. The higher score of SIFT and the lower score of PloyPhen2 indicated that the mutation was more reasonable and rational [23]. To design the mutational sites, first, the AAs formed hydrogen bond with β-lactam molecules were removed from the list of AAs within 5 Å of the active pocket from docking results. Then, the mutations of the remaining AAs were predicted by SIFT software, and the mutational sites with score of 1.0 were screened out. These sites were taken into the PloyPhen2 analysis to select the mutational sites with low scores. Through the sequence alignment of active sites, the AAs involved in a protein binding with drugs also can be applied to mutation.

In addition, basic AAs are easy to form salt bridges with acidic AAs, which can improve the protein stability [5, 20]. Therefore, site-directed mutagenesis can be carried out by analyzing the physicochemical properties of AAs. As BlaR-CTD protein did not contain free cysteine and disulfide bonds, disulfide bond were introduced in the flexible region of the protein to improve the stability by using the software Disulfide by Design 2 (http://cptweb.cpt.wayne.edu/DbD2/). The sites where disulfide bonds could be inserted were based on the predicted energy and B-Factors [11]. The ΣB-factor, which is related to the protein stability, indicates the smearing of atomic electron densities regarding to their equilibrium positions on account of thermal motion and positional disorder [24]. The mutational sites were selected according to the principle of small bonding energy and large ΣB-factor. χ3 torsion angle is defined by rotation of the two Cβ atoms about the Sγ-Sγ bond [6].

### Construction of mutants

The site-directed mutagenesis was used for the structural modification of BlaR-CTD. Mutant plasmids were obtained by one step overlap extension polymerase chain reaction [29], using a recombinant plasmid pET-28a(+)-BlaR-CTD as the template and the primers for the mutation were shown in Table 1. The obtained 0.7-kb DNA fragments were digested with restriction enzymes of *Dpn* I, and the mutant plasmids were transformed into *E*.*coli* DH5α. After confirming by sequencing analysis, the mutant plasmids were transformed into *E*.*coli* BL21 (DE3) for the recombinant expression.

**Table 1:** Primers used for site-directed mutagenesis

Mutation site	Primers	Sequence 5′-3′
A138E	F	CATCCAGCCAATAATTCTCCGGACCTGAGAAATCC
	R	GGATTTCTCAGGTCCGGAGAATTATTGGCTGGATG
Q147K	F	GAGGGGAAATTTTAAGAGAGCC
	R	GGCTCTCTTAAAATTTCCCCTC
I188K	F	GTCCCGGTTTTACCGGATAGTTTTCTGCCATTTGATTCTTCT
	R	AGAAGAATCAAATGGCAGAAAACTATCCGGTAAAACCGGGAC
S190Y	F	AAGTCCCGGTTTTACCGTATAG
	R	TCTATACGGTAAAACCGGGAC
V197D	F	CTCCGTTGATATCTGAAGTCC
	R	GGGACTTCAGATATCAACGG
S19C/G24C		
S19C	F	GATGACTGCACCTTTTTTGATGGCT
	R	GGTGCAGTCATCTTCGTATTCTACA
G24C	F	TTTGATTGCTTCTCAGGAGGTTTTG
	R	AGAAGCAATCAAAAAAGGTGCAGTC
R50C/Q147C		
R50C	F	CCGCCTGTTTCGCACCTGCTTCTAC
	R	GCGAAACAGGCGGTGCTTTCTTTCC
Q147C	F	TCTCTTTGTATTTCCCCTCTTGAAC
	R	GAAATACAAAGAGAGCCATCCAGCC
S76C/ L96C		
S76C	F	CAATTGTCAAATGACGTGGGACGGA
	R	CATTTGACAATTGTTCTTCGTGATGATCCC
L96C	F	AGGATTGTTTCTCTGCGATGAGCAG
	R	GCAGAGAAACAATCCTGGTCTTGAT
S135C/S145C		
S135C	F	ATTTCTGTGGTCCGGCGAATTATTG
	R	CGGACCACAGAAATCCTCATTTCCA
S145C	F	GATGGCTGTCTTCAAATTTCCCCTC
	R	TGAAGACAGCCATCCAGCCAATAAT
E183C/ I188C		
E183C	F	TTAGAATGCTCAAATGGCAGAATTC
	R	TTGAGCATTCTAAACGTATCGAATC
I188C	F	GGCAGATGTCTATCCGGTAAAACC
	R	ATAGACATCTGCCATTTGAGCATTC

Notes: The underline shows the bases corresponding to the mutant amino acids

### Preparation of mutant proteins

*E*. *coli* BL21 (DE3) harboring mutant plasmid were cultured overnight in 1 L of LB broth containing 50 μg·mL^− 1^ kanamycin at 37 °C in shaker. The culture was diluted 100-fold into a fresh LB broth containing 50 μg·mL^− 1^ kanamycin and incubated with vigorous shaking at 37 °C until the OD_600_ reached 0.6. Then 1 mM IPTG was added and the culture was incubated at 18 °C for 12 h. Bacteria were collected by centrifugation at 10,000 rpm for 10 min, and were washed 3 times with ice cold phosphate-buffered saline (PBS) (8 g NaCl, 2.9 g Na_2_HPO_4_·12H_2_O, 0.2 g KCl, 0.2 g KH_2_PO_4_ per liter, pH 7.4). Then, 50 mL binding buffer (7.6 g Na_3_PO_4_, 29.22 g NaCl, 0.68 g imidazole per liter, pH 7.4) was added into the precipitate, and ultrasonic fragmentation was carried out after resuspension of the precipitate. The suspension was centrifuged at 10,000 rpm for 40 min at 4 °C, and the soluble fraction was loaded onto a 2 mL Ni^2+^-charged chelating sepharose resin column. After washing by different concentrations of imidazole eluent, the protein was eluted at imidazole concentration of 60, 100 and 200 mM. The purified mutant proteins were dialyzed against PBS, and were confirmed by SDS-PAGE with Coomassie Brilliant Blue staining and western blot. The protein concentration was determined by the method of Bradford [3].

### Activity assay

The activity assay was based on directly competitive inhibition of binding of horseradish peroxidase-labeled ampicillin (HRP-AMP) to the immobilized BlaR-CTD or its mutants by β-lactams. HRP-AMP was prepared according to the previous study [25]. PBS was used as the coating buffer to dilute the wild-type and mutant proteins, and 100 μL protein (1 μg·mL^− 1^) were coated on the microtiter plates overnight at 4 °C. The plate was washed 3 time with 250 μL of PBST (PBS containing 0.5% Tween-20), followed by blocking with 1% (*w*/*v*) BSA in PBS for 12 h at 4 °C. After washing 3 times with PBST, 50 μl of the standard drug (100 μg·L^− 1^) and 50 μl of HRP-AMP solution were added successively to each well and incubated at 30 °C for 30 min. After washing 3 times with PBST, 100 μl of enzyme substrate TMB was added and incubated at 37 °C for 15 min, and the reaction was stopped by adding 50 μl of 2 M H_2_SO_4_. Then, the absorbance values at 450 nm were measured with microplate reader (Tecan, Seestrasse, Schweiz). The inhibition rate was used to indicate the recognition ability of protein to drugs. The smaller the inhibitions rate the stronger recognition ability.

Inhibition rate (%) = OD value_(drug)_/OD value_(no drug)_ × 100%

### Statistical analyses

Descriptive statistical parameters such as average (**AVG**) and standard deviation (SD) were calculated. Statistical analysis of the data was performed using Microsoft Excel 2003.

## Results

### Homologous model of BlaR-CTD and docking with β-lactams

**Fig. 1:**
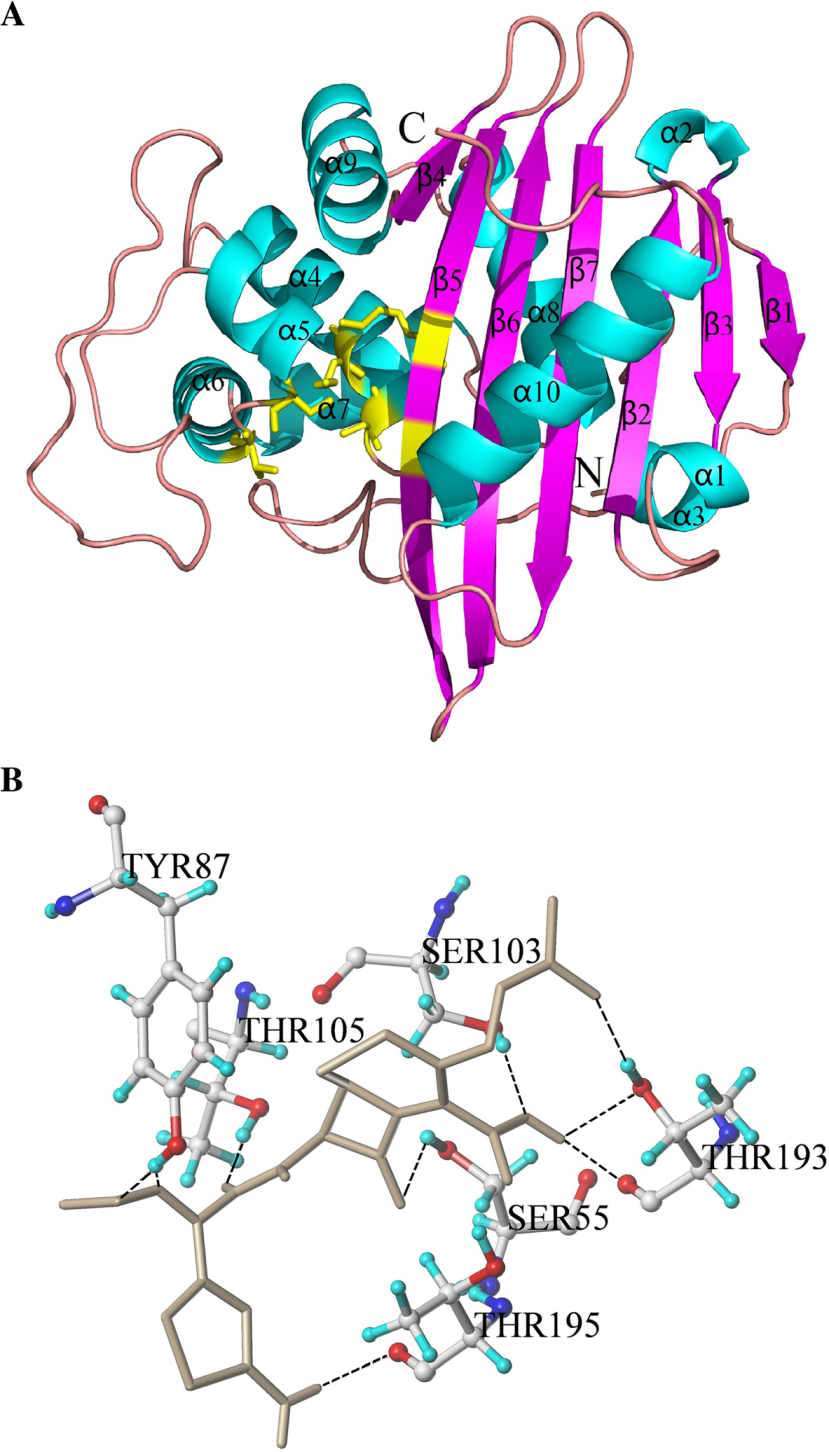
3D structure of BlaR-CTD protein (**a**) and interaction with cefquinome (**b**). **a** The 3D structure of BlaR-CTD was represented as a cartoon, with α-helices colored in cyan, β-strands in magenta, and loops in salmon. The active sites of Ser55, Ser103, Thr105 and Thr195 were colored in yellow. **b** The amino acid residues involved in the interaction of BlaR-CTD with cefquinome. The hydrogen bonds were represented as black dotted lines. The cefquinome molecule was colored in gray. The AAs of the active site were colored in yellow

The 3D structure of BlaR-CTD protein from *B. licheniformis* ATCC14580 was shown in Fig. 1a. It was mainly composed of two domains. One was the α/β domain, which was covered by two α helices (α1 in N-terminal and α10 in C-terminal) on one side of a seven parallel β sheets (β1-β7), with the other side covered by α3 and α8 helix. The other domain was mainly composed of four helices (α5, α6, α7 and α9) encircling the hydrophobic α4 helix. The active pocket of the BlaR-CTD was composed of S(55)TYK(58), S(103)AT(105) and K(192)TG(194) [19].

The docking result of cefquinome with BlaR-CTD was shown in Fig. 1b. Nine hydrogen bonds were formed between the protein and cefquinome in the active pocket. Among the hydrogen bond forming AAs, Ser55, Ser103, Thr105 and Thr193 were reported in the previous literature [19], in which one hydrogen bond was respectively formed with Ser55, Ser103 and Thr105, and three hydrogen bonds were formed with Thr193. In addition, two hydrogen bonds between cefquinome and Tyr87, and one hydrogen bond between the drug and Thr195 were also formed.

Molecular docking between BlaR-CTD protein and other 39 β-lactams was also carried out by Sybyl-X2.0, and 65 key AAs existed within 5 Å around active pocket (Additional file 1: Table S1). The key AAs forming hydrogen bonds to each β-lactam were shown in Table 2, including Ser55, Ser101, Ser102, Ser103, Thr105, Thr193, Thr195, Try87, Glu89, Arg229 and Ser233. When the score was less than 4, the binding ability between protein and drug was poor, and when the score was greater than 7, the binding ability was stronger.

**Table 2:** Binding sites of BlaR-CTD protein to β-lactam antibiotics

β-lactams	The AAs involved in the formation of hydrogen bonds with β-lactams	Score
Pivampicillin	S55, S103, T105, T193, T195	8.35
Moxalactam	S55, S103, T105, T195	7.12
Cefapirin	S55, S103, T105, T193, T195	6.63
Cefalotin	S55, S103, T105, T193, T195	6.59
Ceftazidime	Y87, S103, T195	6.55
Cloxacillin	S55, S103, T105, T195	6.31
Carbenicillin	S55, S103, T193, T195	6.27
Cefoxitin	S55, S103, T105, T193, T195	6.07
Cefamandole	S55, S103, T193, T195	5.96
Floxacillin	S55, Y87, S103, T105, T195	5.88
Cefotaxime	S55, Y87, S103, T105, T193, T195	5.60
Cefepime	E89, S103, T193, T195, S233	5.59
Cefradine	S55, S103, T193, T195	5.51
Cefalonium	S55, S103, T105, T195	5.48
Cefquinome	S55, E89, S103, T193, T195	5.45
Penicillin G	S55, S103, T195	5.43
Cefuroxime	Y87, S101, S103, T105, T193, T195	5.12
Dicloxacillin	S55, S103, T105, T195	5.11
Cefaclor	S55, S103, T193, T195	4.92
Penicillin V	S55, T195	4.70
Cefazolin	S55, S103, T105, T195	4.68
Imipenem	S55, S103, T193, T195	4.65
Ampicillin	S55, S103, T195	4.62
Sulbenicillin	S103, T105, T193, T195	4.53
Ticarcillin	S103, T105, T195	4.52
Cefalexin	E89, S102, T195, S233	4.47
Cefatriaxone	S55, E89, T193, T195, S233	4.37
Methicillin	Y87, T195	4.36
Piperacillin	Y87, T193	4.12
Azlocillin	Y87, T105, T193	3.94
Cefadroxil	S55, S103, T105, T195	3.84
Nafcillin	S55, S103, T105	3.82
Cephalosporin	S103, S233	3.81
Amoxicillin	S55, Y87, T193, T195	3.61
Furbenicillin	S103, T193, T195	3.47
Oxacillin	Y87, T193, T195	3.44
Aztreonam	Y87, E89, S102, T105, T195	3.33
Ceftiofur	S55, T105, T195, S233	2.92
Cefoperazone	S55, E89, S101, S103, T195	2.53
Cefminox	S55, T195, R229	−0.51

### Determination of mutational sites

PBP3 from *Streptococcus pneumoniae* R6 had a high binding ability with cefadroxil, and the docking results of PBP3 and cefadroxil showed that D224 in the active site K(239)TGTTD(244) and cefadroxil formed a hydrogen bond [32]. The sequence alignment demonstrated that K(192)TGTSV(197) in BlaR-CTD activity pocket corresponded to K(239)TGTTD(244) in PBP3. Therefore, the mutation of V197D was selected in order to improve the affinity.

Mutation prediction and evaluation was carried out according to SIFT and Polyphen 2. The mutational sites with a SIFT score of 1.0 were obtained as shown in Additional file 1: Table S2. In order not to affect the structure and function of the protein, the selection of mutational sites of protein design should avoid the active site, especially the hydrogen bond-forming AAs of the protein [4]. Twelve AAs from the list of AAs in the active pocket but exclude the active site and hydrogen bond-forming AAs (Additional file 1: Table S1) showed a SIFT score of 1.0 (Additional file 1: Table S2), and the single point mutations of A138E, Q147K and S190Y with the lowest Polyphen scores were screened out (Additional file 1: Table S3).

In addition, salt bridge and disulfide bond were inserted into the protein flexible region to improve the stability of the protein. Through the analysis of protein structure, I188K mutation could form a salt bridge (2.92 Å) with Glu212 in β6 sheet of BlaR-CTD. According to the prediction by software Disulfide by Design 2.0, the mutant proteins S19C/G24C, R50C/Q147C, S76C/L96C, S135C/S145C, E183C/I188C were selected according to the principle of smaller binding energy value and larger ΣB-factor value as shown in Table 3. In addition, the single point mutation was combined with the disulfide bond insertion mutation when necessary to improve the affinity and stability simultaneously.

**Table 3:** Disulfide bond prediction result of BlaR-CTD using Disulfide by Design 2.0

Location	Residue1		Residue2		Bond		
	Seq #	AA	Seq #	AA	χ3	Energy	ΣB-Factors
Ω-loop							
Flexible region of Ω-loop behind α7	**135**	**SER**	**145**	**SER**	**−94.78**	**2.45**	**120**
Between α helixs and β sheets							
β2 and before the flexible region of α1	**19**	**SER**	**24**	**GLY**	**−68.19**	**3.71**	**113**
Between α3 and β2	28	GLY	47	SER	93.37	2.61	113
β5 and behind the flexible region of α3	54	ALA	194	GLY	−68	8.54	120
Between α4 and β5	56	THR	194	GLY	−106.37	8.44	113
Between α8 and β7	153	GLN	221	ALA	−106.61	4.74	116
Between α5 and α10	195	THR	232	GLY	−100.49	4.36	113
Between β6 and α10	203	HIS	231	ALA	108.1	4.45	113
Between β6 and α10	205	GLY	235	ALA	71.1	3.47	120
Between β6 and α10	207	PHE	235	ALA	−107.6	4.95	113
Between β7 and α10	222	VAL	239	ALA	106.21	4.13	113
Between α helixs							
Behind the flexible region of α3 and α7	**50**	**ARG**	**147**	**GLN**	**−72.34**	**3.89**	**112**
α4 and behind the flexible region of α3	53	PRO	57	TYR	−103.61	3.27	106
α4 and behind the flexible region of α7	57	TYR	141	TRP	−97.51	1.11	106
Between α4 and α9	66	LEU	171	ASN	−96.08	2.58	109
α5 and behind the flexible region of α4	**76**	**SER**	**96**	**LEU**	**−81.41**	**2.69**	**113**
Behind the flexible region of α4	85	TYR	90	TRP	106.13	2.75	106
α5 and behind the flexible region of α4	89	GLU	102	SER	112.89	5.87	109
α5 and behind the flexible region of α4	95	ASP	98	SER	92.89	3.08	106
Between α5 and α6	99	ALA	107	SER	−78.90	2.67	113
α8 and behind the flexible region of α7	149	SER	152	GLU	121.50	3.56	109
Between β sheets							
Between β2 and β7	27	GLY	224	ILE	70.9	6.45	113
Between β4 and β5	**183**	**GLU**	**188**	**ILE**	**110.15**	**2.61**	**116**

Note: The boldface part indicated that the mutant AAs which selected in this study

### Expression and purification of BlaR-CTD mutant proteins

**Fig. 2:**
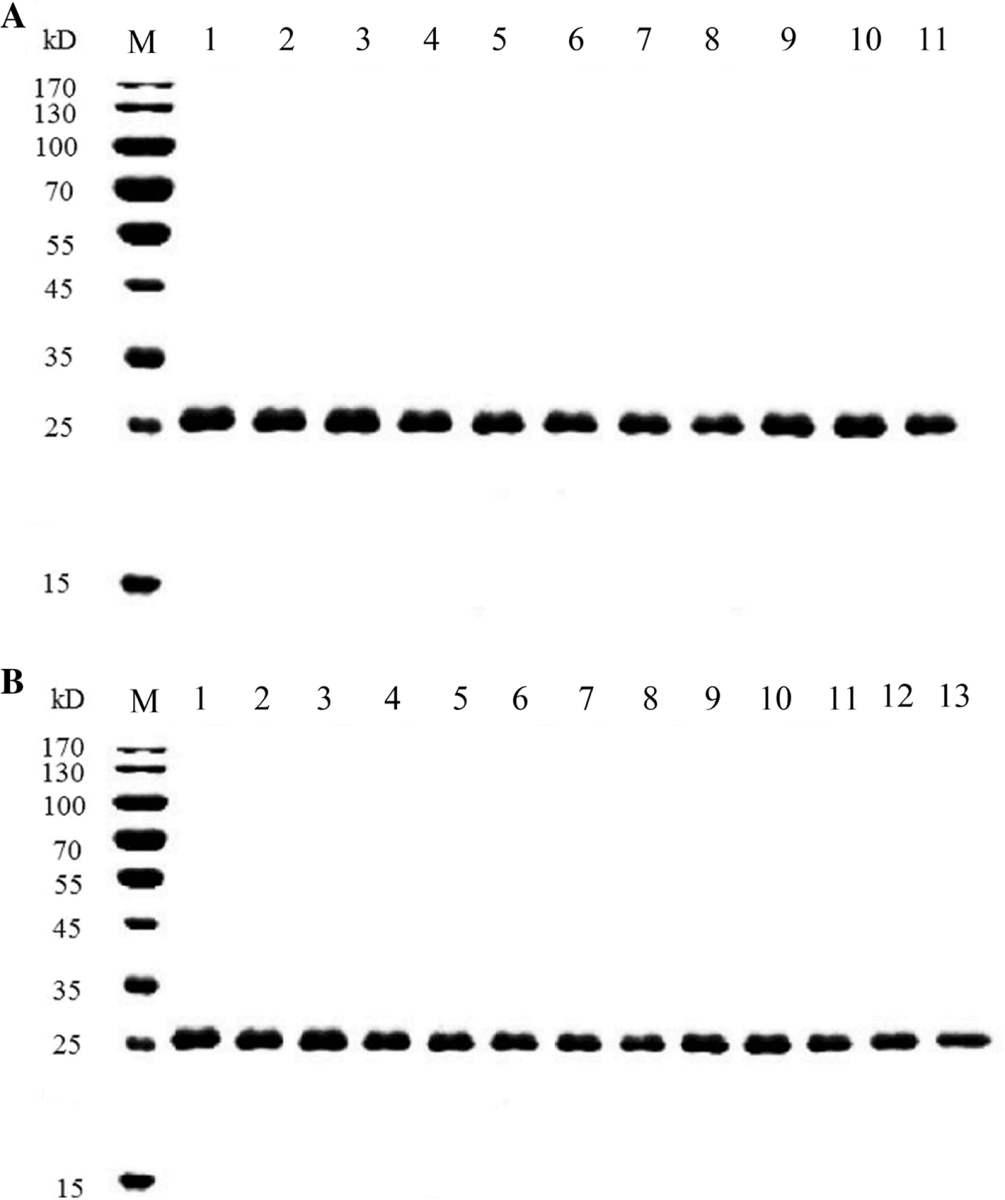
Purification of single point mutants and disulfide bond mutants (**a**) and multipoint mutants of BlaR-CTD protein (**b**). **a** Lanes 1–11 were the purified proteins of BlaR-CTD, A138E, Q147K, I188K, S190Y, V197D, S19C/G24C, R50C/Q147C, S76C/L96C, S135C/S145C, and E183C/I188C, respectively. **b** Lanes 1–13 were the purified proteins of A138E/S19C/G24C, Q147K/S19C/G24C, I188K/S19C/G24C, S190Y/S19C/G24C, V197D/S19C/G24C, A138E/R50C/Q147C, I188K/R50C/Q147C, S190Y/R50C/Q147C, V197D/R50C/Q147C, A138E/E183C/I188C, Q147K/E183C/I188C, S190Y/E183C/I188C, and V197D/E183C/I188C, respectively. M, protein mass marker

The optimized induction condition for the expression of protein was IPTG at final concentration of 1 mM and culturing for 12 h at 18 °C (Additional file 1: Figure S1). As shown in Fig. 2, each single point (Fig. 2a) or combined mutant protein (Fig. 2b) were purified with purity above 95%, and the bands for each mutant were in good agreement with the expected size of 26 kDa. After determination of protein concentration, 600 μg·mL^− 1^ of each purified mutant protein was stored at − 20 °C for further use.

The formation of disulfide bond in mutant proteins (S19C/G24C, R50C/Q147C, S76C/L96C, S135C/S145C, E183C/I188C) were detected by DTNB colorimetry. Standard curve was established according to different absorption values of cysteine at 412 nm (Additional file 1: Figure S2). The absorbance value of mutant protein was measured and the content of cysteine was calculated. The results showed that no free sulfhydryl group was detected in wild type and mutant proteins, indicating the formation of disulfide bond (Additional file 1: Figure S2).

### Activity identification of BlaR-CTD mutant proteins

The binding activities of 5 single point mutation proteins and 5 disulfide bond inserting mutant proteins were shown in Additional file 1: Table S5. The OD values of no free drug competition for mutant proteins S76C/L96C and S135C/S145C were low, indicating that the binding ability of β-lactams of these two mutant proteins was poor, so they were not observed for the following studies.

The inhibition rate, which was inversely proportional to the protein activity, was used to indicate the recognition ability of the protein to the drug compared between each mutant and the wild-type. The results showed that compared with the wild-type protein, the binding ability of each mutant protein to the drug varied, and there was no obvious advantage (Table 4). So, the three active disulfide bond inserting mutants, S19C/G24C, R50C/Q147C and E183C/I188C, and the single point mutations, I188K, V197D, A138E, Q147K, and S190Y, were combined in order to get the superposition effect.

**Table 4:** The inhibition rates (%) of 33 β-lactam drugs on BlaR-CTD proteins of single point mutation and disulfide bond insertion (AVG ± SD, *n* = 5)

Drug	BlaR-CTD	A138E	Q147K	I188K	S190Y	V197D	S19C-G24C	R50C-Q147C	E183C-I188C
Penicillin G	26.2 ± 2.1	**15.3 ± 1.4**	26.7 ± 3.4	25.3 ± 2.8	**21.7 ± 1.6**	34.2 ± 4.2	**17.4 ± 1.5**	34.6 ± 2.5	**20.5 ± 1.4**
Ampicillin	21.2 ± 1.1	21.0 ± 1.8	27.4 ± 2.5	**17.4 ± 1.4**	24.4 ± 2.4	**19.3 ± 1.3**	**18.4 ± 1.3**	**17.5 ± 1.3**	24.7 ± 2.2
Amoxicillin	19.6 ± 1.0	19.3 ± 1.6	28.9 ± 3.4	40.8 ± 3.7	21.7 ± 1.3	25.6 ± 2.8	22.6 ± 3.4	20.4 ± 3.1	16.8 ± 1.3
Nafcillin	29.0 ± 1.4	32.9 ± 4.8	29.5 ± 3.1	34.3 ± 3.5	**24.0 ± 1.9**	35.7 ± 3.3	31.4 ± 2.8	35.2 ± 3.8	26.6 ± 3.7
Oxacillin	23.4 ± 2.3	24.0 ± 3.6	29.3 ± 2.5	26.7 ± 2.9	**22.3 ± 2.5**	25.0 ± 3.4	26.6 ± 2.1	21.5 ± 2.4	23.6 ± 2.4
Azlocillin	15.2 ± 1.1	**10.9 ± 1.7**	14.7 ± 1.4	**10.6 ± 1.3**	16.7 ± 1.7	17.0 ± 1.6	14.1 ± 1.3	**12.8 ± 1.2**	24.9 ± 4.3
Penicillin V	15.5 ± 1.5	**12.7 ± 1.2**	**7.5 ± 1.0**	16.7 ± 1.1	**10.9 ± 1.2**	**12.2 ± 1.2**	25.9 ± 2.6	35.2 ± 4.6	16.4 ± 2.9
Piperacillin	6.5 ± 0.9	8.0 ± 1.0	12.0 ± 1.6	6.2 ± 0.8	27.3 ± 2.4	20.3 ± 3.6	13.2 ± 1.5	12.2 ± 1.5	10.4 ± 1.1
Dicloxacillin	27.5 ± 2.1	30.3 ± 2.9	**23.2 ± 3.4**	**24.9 ± 2.4**	31.7 ± 4.2	25.0 ± 4.5	31.6 ± 5.1	**24.2 ± 2.0**	26.1 ± 1.3
Cloxacillin	21.1 ± 1.8	25.5 ± 2.4	26.5 ± 3.1	44.4 ± 5.1	22.0 ± 2.5	25.3 ± 3.2	20.2 ± 1.3	31.7 ± 4.7	22.5 ± 3.4
Cefalotin	26.3 ± 2.4	24.6 ± 2.5	27.0 ± 1.9	24.7 ± 2.6	**20.9 ± 1.7**	34.0 ± 5.1	**18.6 ± 1.2**	**18.4 ± 1.3**	**16.7 ± 1.2**
Cefoperazone	21.5 ± 1.3	**18.3 ± 1.7**	29.1 ± 4.1	25.8 ± 2.7	30.5 ± 4.2	20.5 ± 3.6	**16.2 ± 1.3**	25.5 ± 2.1	31.5 ± 4.6
Cefazolin	32.0 ± 2.9	**26.1 ± 2.3**	30.5 ± 3.2	29.8 ± 3.1	**23.4 ± 3.4**	**26.9 ± 3.4**	40.1 ± 2.5	31.5 ± 4.5	40.2 ± 5.1
Cefalexin	88.4 ± 6.8	87.7 ± 9.4	81.9 ± 7.6	**69.9 ± 4.5**	**65.2 ± 7.5**	**75.2 ± 10.1**	**63.5 ± 5.4**	**75.8 ± 9.1**	**62.5 ± 9.3**
Cefatriaxone	25.5 ± 1.5	**19.7 ± 1.2**	24.8 ± 4.8	23.5 ± 2.4	**19.4 ± 1.4**	**21.2 ± 1.8**	**17.3 ± 1.2**	31.4 ± 2.7	**20.5 ± 1.2**
Cefadroxil	69.8 ± 4.8	70.6 ± 8.1	65.2 ± 5.6	**59.6 ± 6.1**	69.9 ± 8.5	**56.7 ± 5.2**	84.9 ± 10.5	**55.2 ± 3.4**	71.5 ± 7.9
Ceftiofur	21.4 ± 2.6	**16.7 ± 1.5**	25.7 ± 2.4	34.6 ± 3.2	28.6 ± 3.4	19.4 ± 1.4	20.6 ± 4.2	22.1 ± 1.5	25.1 ± 3.4
Cefepime	9.4 ± 0.8	17.7 ± 1.2	27.9 ± 2.8	21.7 ± 1.6	17.4 ± 1.8	18.0 ± 1.6	16.9 ± 1.6	14.5 ± 1.1	22.4 ± 2.6
Cefaclor	19.7 ± 1.4	18.5 ± 2.4	31.5 ± 3.4	20.9 ± 1.2	19.6 ± 1.6	17.4 ± 1.2	**14.8 ± 2.1**	**15.8 ± 2.1**	20.6 ± 1.3
Cefquinome	19.5 ± 1.2	**11.8 ± 1.5**	**16.9 ± 1.4**	23.9 ± 2.5	**12.0 ± 1.3**	**16.6 ± 1.4**	**11.3 ± 1.2**	**16.8 ± 1.3**	19.9 ± 1.2
Cefradine	61.2 ± 5.7	59.6 ± 6.3	67.3 ± 7.3	58.7 ± 5.6	72.0 ± 5.7	56.1 ± 7.1	**49.9 ± 6.1**	**52.4 ± 4.5**	68.4 ± 8.2
Cefapirin	19.6 ± 1.3	**14.1 ± 1.2**	29.1 ± 1.7	23.3 ± 1.3	**10.4 ± 1.0**	**15.9 ± 1.9**	**8.3 ± 0.9**	18.5 ± 3.1	22.4 ± 3.7
Cefuroxime	45.8 ± 5.3	49.5 ± 5.4	46.2 ± 3.1	52.4 ± 6.1	46.2 ± 6.1	43.6 ± 4.1	46.8 ± 5.2	45.1 ± 4.1	43.5 ± 5.1
Moxalactam	21.4 ± 2.9	19.5 ± 1.7	32.1 ± 4.5	23.4 ± 3.2	**13.3 ± 1.2**	22.1 ± 2.3	**8.2 ± 1.0**	20.4 ± 2.5	44.2 ± 3.1
Cefalonium	5.2 ± 0.7	6.2 ± 0.8	11.7 ± 1.6	6.3 ± 0.8	21.5 ± 3.3	32.9 ± 4.3	10.3 ± 1.3	14.2 ± 1.3	15.2 ± 1.5
Cefminox	19.0 ± 1.6	22.9 ± 3.4	18.0 ± 1.2	17.4 ± 1.5	**12.8 ± 1.2**	20.8 ± 3.4	**16.2 ± 2.1**	25.9 ± 3.0	18.5 ± 1.1
Ceftazidime	23.1 ± 2.6	**19.8 ± 1.5**	**16.8 ± 2.3**	**19.6 ± 2.1**	21.3 ± 2.3	**19.3 ± 1.8**	21.1 ± 1.9	**18.5 ± 1.2**	24.8 ± 3.1
Carbenicillin	6.7 ± 0.8	16.8 ± 1.1	15.2 ± 1.3	11.1 ± 1.0	19.6 ± 1.4	22.5 ± 2.0	15.0 ± 1.2	17.7 ± 1.1	15.9 ± 1.6
Cefoxitin	12.7 ± 2.6	17.0 ± 1.3	19.9 ± 1.2	16.0 ± 1.4	10.5 ± 2.1	11.7 ± 1.8	12.3 ± 2.1	11.5 ± 1.8	15.5 ± 1.0
Floxacillin	6.2 ± 0.8	7.8 ± 1.0	12.3 ± 1.3	8.7 ± 1.1	**4.3 ± 0.5**	7.2 ± 1.3	10.3 ± 1.3	20.4 ± 3.3	16.7 ± 2.1
Sulbenicillin	8.7 ± 1.1	12.5 ± 1.1	16.6 ± 2.5	10.0 ± 1.0	18.3 ± 1.7	25.5 ± 1.2	**7.2 ± 0.9**	13.5 ± 2.1	15.2 ± 1.3
Ticarcillin	12.5 ± 1.6	13.8 ± 2.4	15.4 ± 1.4	12.3 ± 2.6	27.9 ± 3.5	24.9 ± 2.1	15.1 ± 1.5	11.3 ± 1.3	11.4 ± 2.0
Aztreonam	93.1 ± 6.7	86.8 ± 5.7	95.4 ± 10.1	90.0 ± 9.4	94.3 ± 10.1	100.9 ± 9.7	106.4 ± 9.1	100.3 ± 9.0	97.5 ± 9.5

Note: The statistical method was used to analyze the significant difference between the wild-type protein and mutant proteins. The underlined number indicated that the inhibition rate was significantly increased, and the boldface number showed that the inhibition rate was significantly reduced (*p* < 0.05)

Among the multipoint mutation proteins, three mutant proteins with highest affinity, I188K/S19C/G24C, A138E/R50C/Q147C, and S190Y/E183C/I188C, exhibited significantly lower inhibition rate to 33, 22 and 21 drugs respectively than the wild-type protein (Table 5).

**Table 5:** The inhibition rate (%) of 33 β-lactam drugs on BlaR-CTD proteins of multipoint mutations (AVG ± SD, *n* = 5)

Drug	BlaR-CTD	A138E/S19C/G24C	Q147K/S19C/G24C	I188K/S19C/G24C	S190Y/S19C/G24C	V197D/S19C/G24C	A138E/R50C/Q147C	S190Y/R50C/Q147C	V197D/R50C/Q147C	I188K/R50C/Q147C	A138E/E183C/I188C	Q147K/E183C/I188C	S190Y/E183C/I188C	V197D/E183C/I188C
Penicillin G	23.7 ± 3.5	**18.5 ± 1.2**	45.3 ± 4.5	**7.4 ± 1.3**	34.0 ± 3.3	32.2 ± 4.4	**7.6 ± 1.0**	36.5 ± 5.6	67.8 ± 7.9	**10.4 ± 2.0**	**8.4 ± 1.0**	**8.9 ± 0.7**	**7.4 ± 1.1**	47.1 ± 4.3
Ampicillin	28.2 ± 2.6	**17.6 ± 3.4**	**5.1 ± 1.1**	**7.5 ± 1.4**	**10.9 ± 1.3**	**16.8 ± 1.7**	37.3 ± 5.1	**18.3 ± 2.3**	**10.6 ± 1.2**	**11.2 ± 2.1**	**14.0 ± 2.2**	**6.8 ± 1.1**	**7.2 ± 1.4**	**10.2 ± 1.4**
Amoxicillin	21.2 ± 2.4	20.5 ± 3.4	**5.3 ± 0.6**	**12.6 ± 2.4**	**8.8 ± 1.2**	**16.5 ± 1.1**	**11.6 ± 1.3**	21.6 ± 2.7	**12.3 ± 1.5**	**11.0 ± 1.3**	**8.6 ± 1..3**	**6.7 ± 0.6**	**7.2 ± 1.0**	**10.0 ± 1.2**
Nafcillin	28.9 ± 3.7	27.9 ± 4.3	**14.9 ± 0.7**	**18.4 ± 2.1**	31.1 ± 2.5	**14.6 ± 2.3**	**13.1 ± 2.0**	26.5 ± 3.5	**12.1 ± 1.2**	**17.8 ± 2.5**	**13.0 ± 2.5**	**9.6 ± 0.7**	**8.3 ± 1.2**	**15.3 ± 1.1**
Oxacillin	24.0 ± 1.8	22.5 ± 2.3	**5.9 ± 0.6**	**16.6 ± 2.3**	**10.4 ± 1.3**	24.5 ± 3.5	**11.7 ± 2.1**	28.8 ± 3.4	**13.5 ± 2.3**	**12.8 ± 1.6**	**9.6 ± 1.0**	**7.2 ± 1.0**	**7.0 ± 1.3**	26.8 ± 3.5
Azlocillin	19.7 ± 2.5	**12.0 ± 1.1**	**15.2 ± 1.1**	**11.6 ± 2.4**	28.6 ± 3.4	**12.6 ± 1.7**	19.0 ± 1.3	19.7 ± 1.2	20.5 ± 3.5	**8.9 ± 0.7**	**7.4 ± 1.0**	**5.5 ± 1.3**	**5.7 ± 0.9**	**11.2 ± 1.2**
Penicillin V	24.7 ± 2.6	**20.1 ± 1.4**	**5.2 ± 1.2**	**16.3 ± 2.2**	29.4 ± 2.3	26.6 ± 3.4	**17.9 ± 3.1**	24.8 ± 4.5	**12.6 ± 2.3**	**9.6 ± 1.0**	**7.8 ± 0.8**	**5.7 ± 1.0**	**6.0 ± 0.8**	**11.5 ± 2.4**
Piperacillin	12.8 ± 3.1	24.0 ± 4.2	14.4 ± 4.1	**9.2 ± 1.2**	17.5 ± 2.3	15.1 ± 2.2	14.3 ± 1.3	12.8 ± 2.3	16.3 ± 1.2	**8.9 ± 1.3**	**7.1 ± 1.4**	**5.6 ± 1.1**	**6.0 ± 1.0**	12.1 ± 1.2
Dicloxacillin	24.3 ± 4.2	26.2 ± 1.3	**4.8 ± 0.7**	**11.6 ± 2.4**	29.0 ± 3.2	26.6 ± 1.8	24.5 ± 5.1	23.2 ± 3.5	**7.0 ± 0.9**	**11.7 ± 2.3**	**9.2 ± 0.6**	**6.7 ± 0.5**	**8.1 ± 0.7**	21.2 ± 3.4
Cloxacillin	23.6 ± 2.4	**18.5 ± 2.4**	**15.2 ± 2.3**	**10.2 ± 4.2**	29.4 ± 2.4	28.3 ± 3.4	**14.2 ± 0.8**	24.2 ± 2.7	24.3 ± 2.3	**12.0 ± 2.1**	**9.6 ± 1.5**	**7.0 ± 1.2**	**6.7 ± 1.0**	**10.9 ± 1.1**
Cefalotin	17.3 ± 1.5	**13.0 ± 3.4**	24.8 ± 3.4	**8.6 ± 0.9**	**9.2 ± 1.1**	**14.3 ± 2.2**	**14.2 ± 1.0**	**8.1 ± 1.0**	14.8 ± 2.2	**11.6 ± 2.0**	**9.9 ± 1.1**	**6.7 ± 1.1**	**6.4 ± 1.1**	38.7 ± 4.7
Cefoperazone	20.3 ± 2.4	**8.7 ± 1.6**	**9.9 ± 1.1**	**6.2 ± 1.0**	27.7 ± 3.3	18.8 ± 1.1	**5.9 ± 0.4**	**8.6 ± 0.8**	**7.3 ± 1.1**	21.6 ± 3.1	23.2 ± 3.7	**7.7 ± 1.4**	22.5 ± 1.3	**9.2 ± 1.6**
Cefazolin	31.3 ± 4.6	**15.9 ± 2.3**	**20.8 ± 3.3**	**10.1 ± 2.6**	**21.4 ± 2.6**	**19.6 ± 2.3**	**12.1 ± 3.3**	47.6 ± 6.7	**19.1 ± 1.2**	33.0 ± 2.4	**21.2 ± 1.8**	**20.7 ± 2.2**	**13.7 ± 2.2**	**19.4 ± 2.2**
Cefalexin	80.1 ± 9.7	83.3 ± 9.4	**65.6 ± 7.9**	**63.5 ± 6.7**	**65.9 ± 5.9**	80.1 ± 7.9	79.7 ± 6.4	**38.4 ± 5.8**	85.0 ± 9.9	91.5 ± 9.4	69.8 ± 5.5	69.0 ± 7.8	**66.6 ± 7.9**	77.4 ± 8.9
Cefatriaxone	22.1 ± 4.6	22.5 ± 2.3	26.5 ± 3.2	**10.9 ± 1.0**	41.9 ± 6.7	61.7 ± 7.7	**15.0 ± 2.5**	**12.6 ± 1.3**	29.5 ± 3.4	37.2 ± 4.6	26.6 ± 4.0	17.7 ± 2.3	**10.2 ± 1.5**	29.2 ± 3.4
Cefadroxil	70.2 ± 5.5	63.8 ± 5.4	**58.8 ± 4.6**	**45.1 ± 6.1**	**56.3 ± 4.5**	68.3 ± 5.6	**59.8 ± 6.7**	**29.4 ± 3.5**	76.0 ± 8.9	81.3 ± 7.6	**51.0 ± 7.6**	**50.4 ± 5.5**	**37.7 ± 4.3**	66.2 ± 7.8
Ceftiofur	16.1 ± 1.1	**10.6 ± 1.7**	15.2 ± 2.1	**7.6 ± 0.8**	**12.0 ± 1.3**	26.8 ± 1.5	**8.8 ± 1.2**	**10.1 ± 1.2**	**14.2 ± 1.2**	26.3 ± 4.8	24.2 ± 2.1	16.4 ± 1.6	**8.3 ± 1.1**	15.9 ± 3.4
Cefepime	13.2 ± 3.3	**9.5 ± 1.1**	13.2 ± 1.3	**6.0 ± 1.0**	18.3 ± 1.1	13.0 ± 4.7	**7.1 ± 0.9**	**8.3 ± 1.1**	**8.9 ± 1.0**	25.2 ± 2.2	26.7 ± 3.7	20.3 ± 2.2	16.6 ± 2.4	**8.2 ± 0.7**
Cefaclor	19.2 ± 2.2	32.6 ± 2.3	17.1 ± 3.4	**8.1 ± 1.4**	28.3 ± 2.7	38.4 ± 2.2	**13.8 ± 2.1**	**13.0 ± 1.3**	26.8 ± 3.4	40.2 ± 4.3	36.8 ± 4.6	31.4 ± 3.4	**15.5 ± 2.3**	28.9 ± 2.3
Cefquinome	21.1 ± 1.4	**12.6 ± 2.2**	**4.4 ± 1.1**	**6.3 ± 0.7**	21.5 ± 1.4	**14.1 ± 1.3**	**6.4 ± 1.0**	**6.5 ± 0.8**	27.8 ± 2.2	25.3 ± 3.4	23.1 ± 2.1	**16.8 ± 1.2**	**16.8 ± 1.6**	29.0 ± 4.5
Cefradine	59.6 ± 6.9	66.5 ± 8.9	68.0 ± 7.8	**43.1 ± 8.7**	51.8 ± 6.7	58.1 ± 8.9	74.2 ± 5.9	69.1 ± 5.2	65.4 ± 8.3	74.1 ± 6.4	58.7 ± 8.9	76.1 ± 7.6	74.6 ± 8.6	53.3 ± 8.9
Cefapirin	13.5 ± 3.4	10.2 ± 2.3	**5.6 ± 1.1**	**5.8 ± 1.1**	11.2 ± 1.7	14.9 ± 1.1	**6.4 ± 1.1**	**6.9 ± 0.9**	**9.3 ± 1.4**	22.2 ± 2.5	15.3 ± 2.4	16.0 ± 3.3	13.2 ± 1.5	**7.4 ± 1.2**
Cefuroxime	44.3 ± 5.6	47.4 ± 5.6	49.1 ± 6.5	**30.0 ± 4.5**	68.9 ± 5.5	57.1 ± 4.5	**36.1 ± 3.5**	**20.5 ± 2.4**	46.3 ± 3.4	39.2 ± 6.7	**31.4 ± 3.8**	**26.0 ± 2.2**	**29.5 ± 3.6**	**33.1 ± 4.6**
Moxalactam	20.0 ± 1.3	**10.6 ± 1.5**	**14.9 ± 4.2**	**7.4 ± 0.8**	29.6 ± 4.3	21.2 ± 3.4	29.0 ± 3.3	**8.1 ± 1.0**	**12.0 ± 1.1**	18.8 ± 2.7	18.4 ± 4.5	**16.5 ± 2.7**	17.9 ± 2.4	28.7 ± 3.4
Cefalonium	8.9 ± 1.1	7.6 ± 2.1	14.4 ± 1.1	**6.5 ± 1.5**	30.8 ± 5.4	28.0 ± 2.3	**5.1 ± 1.0**	**6.6 ± 0.8**	**6.5 ± 0.8**	19.8 ± 3.8	19.3 ± 1.2	14.8 ± 2.6	12.3 ± 3.3	9.9 ± 1.5
Cefminox	25.5 ± 3.6	42.4 ± 3.8	48.7 ± 6.9	**20.2 ± 3.3**	67.2 ± 7.5	66.9 ± 7.8	24.5 ± 4.5	**17.5 ± 2.4**	79.0 ± 5.8	24.8 ± 3.3	**17.6 ± 3.3**	22.5 ± 3.4	25.0 ± 4.1	82.0 ± 9.2
Ceftazidime	20.2 ± 4.4	**10.8 ± 1.2**	15.9 ± 3.4	**8.1 ± 1.0**	33.3 ± 3.6	37.2 ± 3.2	**8.6 ± 2.2**	**9.1 ± 1.0**	**14.9 ± 2.2**	21.7 ± 2.7	21.4 ± 3.2	17.4 ± 1.2	**12.0 ± 2.3**	**13.4 ± 1.3**
Carbenicillin	8.9 ± 1.1	**6.1 ± 1.1**	15.3 ± 4.2	**6.8 ± 0.8**	26.6 ± 4.5	30.1 ± 4.4	**6.1 ± 1.1**	7.4 ± 1.2	9.2 ± 1.6	10.0 ± 1.9	21.3 ± 2.4	21.7 ± 3.3	8.5 ± 0.9	8.1 ± 0.8
Cefoxitin	14.9 ± 2.2	**9.3 ± 0.7**	22.9 ± 2.2	**9.7 ± 1.7**	52.4 ± 6.7	40.6 ± 6.6	16.2 ± 3.4	12.2 ± 2.3	25.0 ± 3.5	**9.2 ± 2.4**	**9.6 ± 1.0**	**10.2 ± 1.1**	12.8 ± 2.2	14.0 ± 2.4
Floxacillin	6.3 ± 0.8	5.4 ± 0.9	15.0 ± 1.3	**4.7 ± 1.3**	33.8 ± 4.2	26.1 ± 6.3	5.6 ± 0.8	6.6 ± 1.1	7.5 ± 1.1	17.6 ± 1.3	13.1 ± 1.6	12.2 ± 2.6	6.8 ± 1.4	6.9 ± 1.1
Sulbenicillin	18.9 ± 2.3	**10.4 ± 1.3**	15.2 ± 2.1	**7.2 ± 0.8**	32.9 ± 3.7	32.7 ± 2.4	**8.1 ± 1.1**	**7.9 ± 1.2**	**12.6 ± 2.3**	20.6 ± 3.2	18.7 ± 3.3	**13.3 ± 3.7**	14.8 ± 3.3	**9.3 ± 1.4**
Ticarcillin	19.7 ± 1.7	**10.5 ± 1.5**	17.3 ± 3.4	**8.8 ± 1.3**	36.2 ± 5.4	30.9 ± 3.2	**8.1 ± 1.6**	**8.0 ± 1.0**	**15.4 ± 1.2**	16.9 ± 2.7	**15.2 ± 1.2**	**14.8 ± 2.2**	**14.4 ± 2.7**	**10.2 ± 1.0**
Aztreonam	102.5 ± 9.1	95.7 ± 10.4	100.1 ± 9.3	**90.3 ± 6.6**	**62.5 ± 6.8**	**70.2 ± 6.5**	105.6 ± 9.1	**65.5 ± 8.2**	**74.3 ± 8.2**	102.5 ± 9.9	89.6 ± 9.7	98.6 ± 7.8	95.0 ± 9.3	**85.0 ± 5.3**

Note: The statistical method was used to analyze the significant difference between the wild-type protein and mutant proteins. The underlined number indicated that the inhibition rate was significantly increased, and the boldface number showed that the inhibition rate was significantly reduced (*p* < 0.05

### Stability analysis of BlaR-CTD mutant proteins

**Fig. 3:**
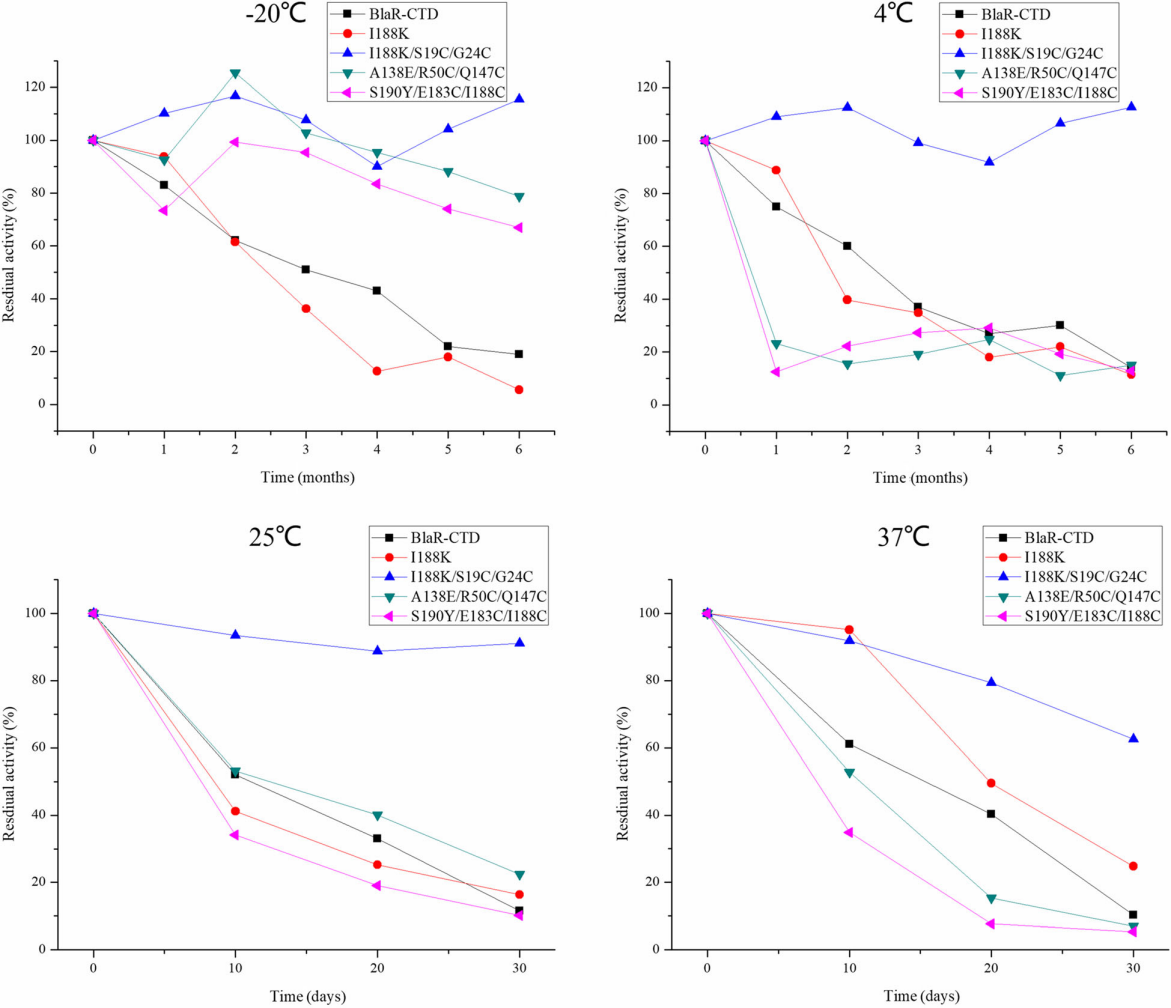
Stability of BlaR-CTD and mutant proteins. The wild-type BlaR-CTD protein and its mutants, I188K I188K/S19C/G24C, A138E/R50C/Q147C and S190Y/E183C/I188C were respectively stored at − 20 °C and − 4 °C for 6 months and at 25 °C and 37 °C for 30 days. At indicated times, each protein was taken to react with HRP-AMP. Residual activity (%) = OD value _(after storage)_/ OD value _(before storage)_ × 100%

By comparing the stability of the mutant and wild-type protein, the stability of I188K/S19C/G24C, A138E/R50C/Q147C and S190Y/E183C/I188C were higher than that of wild-type protein and single point mutant protein I188K at − 20 °C in 6 months (Fig. 3). At 4 °C, I188K/S19C/G24C was more stable than wild-type protein and mutant proteins I188K, A138E/R50C/Q147C and S190Y/E183C/I188C for 6 months. At 25 °C and 37 °C, the stability of mutant protein I188K/S19C/G24C was higher than that of wild-type protein and mutant proteins I188K, A138E/R50C/Q147C and S190Y/E183C/I188C in 30 days. When the mutant proteins A138E/R50C/Q147C and S190Y/E183C/I188C were stored at 4 °C, 25 °C and 37 °C for 1 month, the protein activity decreased to about 20%, and these two mutant proteins only has a good stability at − 20 °C.The activity of I188K/S19C/G24C could maintain over 90% in 6 months at − 20 °C and 4 °C, and over 90 and 60% at 25 °C and 37 °C respectively in 30 days (Fig. 3).

### Comparison of protein structure and binding mode of I188K/S19C/G24C with the wild-type BlaR-CTD protein

**Fig. 4:**
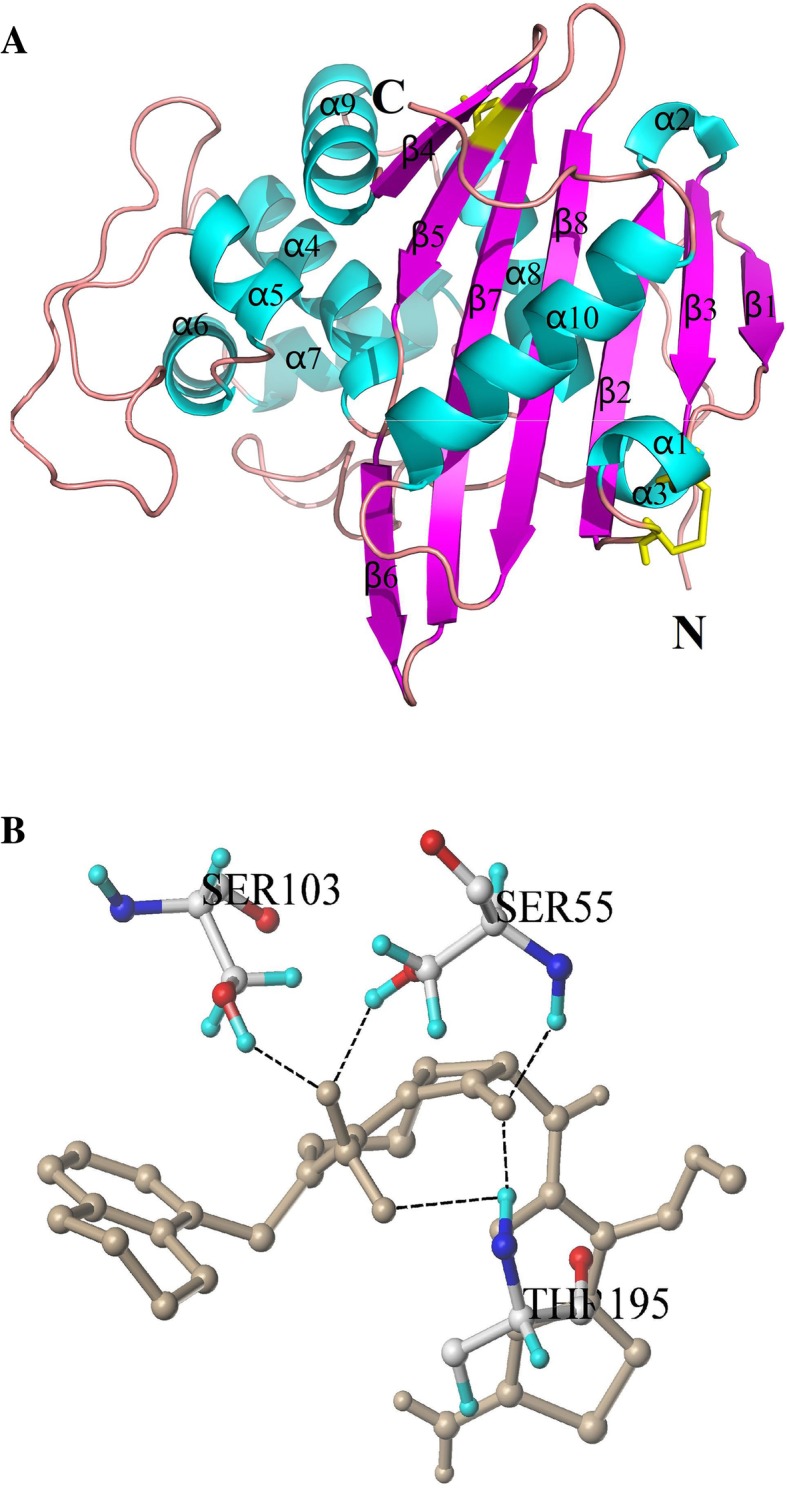
3D structure of mutant protein I188K/S19C/G24C (**a**) and interaction with cefquinome (**b**). **a** The 3D structure of BlaR-CTD was represented as a cartoon, with α-helices colored in cyan, β-strands in magenta, and loops in salmon. The mutational sites of I188K, S19C and G24C were colored in yellow. **b** The AA residues involved in the interaction of protein with cefquinome. The hydrogen bonds were represented as black dotted lines. The cefquinome molecule was colored in gray. The AAs of the mutational sites were colored in yellow

According to the 3D structure of BlaR-CTD protein obtained earlier (Fig. 1a),

the 3D structure of mutant protein I188K/S19C/G24C was obtained by homologous modelling of Sybyl-X2.0 software (Fig. 4a). The β5 sheet in the α/β domain of the wild-type became two β sheets, β5 and β6, in the mutant protein I188K/S19C/G24C because of I188K mutation. The active site, K(192)TG(194), changed from β sheet in the wild-type to random coil in the mutant protein, which may have effect on the affinity of the protein. Sybyl-X2.0 was used for the molecular docking between mutant protein I188K/S19C/G24C and β-lactams. As shown in Fig. 4b, five hydrogen bonds were formed between Ser55, Ser103, Thr195 in the mutant protein and cefquinome, among which two hydrogen bonds formed with Ser55 and Thr195 respectively, and one hydrogen bond formed with Ser103. Hydrogen bond is the strongest non-covalent interactions, and the more hydrogen bonds, the stronger the binding ability of the protein to the ligand [28]. However, compared with the wild-type protein (Fig. 1b), the number of hydrogen bonds was decreased 44.4% in I188K/S19C/G24C docked with cefquinome (from 9 hydrogen bonds in the wild-type to 5 hydrogen bonds in I188K/S19C/G24C) (Fig. 4b) while the binding activity improved more than 3 fold (Table 5). The key AAs involved in the formation of hydrogen bonds between protein and 40 β-lactam drug were obtained (Additional file 1: Table S6). In a comparison of the wild type protein result (Table 2), it was found that the total score of the mutant protein was improved as a whole.

## Discussion

### Evaluation of the affinity of BlaR-CTD mutant proteins

The recombinant BlaR-CTD protein originating from *B. licheniformis* ATCC 14580 and heterologously expressed in *E. coli* contained a total of 288 residues, corresponding to a theoretical molecular mass of 32,427 Da, since it contained an N-terminal extension sequence originating from pET28 vector (Additional file 1: Figure S3, amino acid residue numbers from − 34 to − 1). However, the molecular weight of the recombinant BlaR-CTD protein estimated from SDS-PAGE was only 26 kDa (Fig. 2). We attribute this discrepancy to the fact that due to the low isoelectric point of BlaR-CTD (pI = 5.24) in the alkaline separation gel the denatured protein might be more negatively charged than average proteins, accelerating its mobility. Similarly, in the study of a related 262 residues protein (Met346-Arg601 C-terminal domain of the BlaR sensory-transducer protein of B. licheniformis with a hexapeptide extension), the recombinant protein with 29.3 kDa theoretical molecular mass and pI 5.72 migrated with 26 kDa on SDS-PAGE [17].

The affinity of BlaR-CTD to each β-lactam is different, due to the different chemical structures of drugs, the interaction and binding modes of BlaR-CTD protein with drugs are also different. There are some difficulties in selecting mutation sites because it was uncertain which amino acids in proteins interact directly with drugs. Molecular docking was used to find the key amino acids that may interact with each drug (e.g., those amino acids forming hydrogen bonds with the drug). Through the results of molecular docking between protein and β-lactam antibiotics, the amino acid sites involved in the hydrogen bonding between protein and drug were obtained. These key amino acids should be avoided in the selection of mutation sites, which provided a reference for the selection of subsequent mutation sites.

Mutation of V197D in BlaR-CTD was designed based on the sequence alignment with PBP3 in which D224 formed a hydrogen bond with cefadroxil [32]. The inhibition rate of V197D to cefadroxil was significantly decreased in this study (Table 4), indicating that V197D had a higher affinity for cefadroxil, which was consistent with the result of PBP3 to cefadroxil [32]. This suggests that it is reliable to select mutation through AA sequence as compared to active sites. The inhibition rate of mutant protein Q147K on 17 drugs increased significantly. The mutational site Gln147 is located in the flexible region between α7 and α8, and the distance between Arg50 and Gln147 (4.38 Å) is close in 3D structure. When the polar Gln147 with no charge was changed into the alkaline Lys147 with positive charge, it was easy to produce the repulsion effect since both Arg50 and Lys147 had a positive charge and the side chain was large, making the active pocket become smaller, thus leading to the decrease of protein affinity.

Among the disulfide bond inserting mutants, S76C/L96C and S135C/S145C exhibited nearly no activity (Additional file 1: Table S5). Since the mutational site Leu96 in the mutant protein S76C/L96C was located within 5 Å of the active pocket (Additional file 1: Table S1), its mutation may influence the protein activity pocket, resulting in the reduced activity of mutant protein.

Compared with S19C-G24C mutant protein (Table 4), the inhibition rate of mutant protein A138E/S19C/G24C and I188K/S19C/G24C decreased significantly (Table 5), indicating a superposition effect. But the inhibition rate of other combined mutant proteins (Q147K/S19C/G24C, S190Y/S19C/G24C, V197D/S19C/G24C) was not quite different from that of single point mutation. It might be due to the mutation of hydrophobic AAs to polar AAs, which increased the affinity of the proteins. The mutation of V197D was also from hydrophobic AA to polar AA, but Asp197 was an acid AA, and it was easy to form a salt bridge with the alkaline residue Arg229, which was close to it, thus making the active pocket smaller and reducing the protein activity. Compared with R50C-Q147C (Table 4), the inhibition rate of the A138E/R50C/Q147C to 21 drugs significantly decreased (Table 5). It probably due to Glu138 and Cys147 were located in the flexible region of Ω-loop behind α7, and the spatial locations are relatively close (13.35 Å), resulting in the change in the spatial structure of the protein and the increase of the affinity. The inhibition rates of combined mutations of A138E, Q147K, S190Y, and V197D with E183C-I188C to penicillins were significantly reduced, while the inhibition rates to most of the cephalosporins were significantly increased (Table 5). Cys183 was located on β4, Cys188 was located on β5, and they were located between adjacent β sheets (the distance is 4.29 Å), which may lead to a smaller protein active pocket. The β-lactam ring of cephalosporins is larger than that of penicillin [27]. When cephalosporins are entering into mutant proteins, the binding ability of the proteins may be reduced due to the small space of active pocket. The inhibitory rate of the mutant protein S190Y/E183C/I188C on the 21 drugs decreased significantly (Table 5). The spatial distances between Ser190, Glu183 and Ile188 were very close. The protein structure changed correspondingly after the mutation, since Tyr is larger than Ser, and the affinity of the protein was improved. Moreover, it is interesting to note that Tyr190 and Cys188 are located in the β5, implicating that this region is important for the binding ability of protein to drug.

The total score of molecular docking which predicted by software represented the binding affinity between protein and drug, and the score was expressed as binding constant, pKd [13]. A high value of total score indicates a good affinity of the drug with protein. Among the docking results for 40 drugs, the docking scores of I188K/S19C/G24C with 23 drugs were increased compared with those of the wild-type (Additional file 1: Table S6). In the 33 drugs involved in the activity test, the docking scores between the mutant protein and 18 drugs were increased, but the mutant protein showed a higher affinity to all of the 33 drugs (Table 5). Since the molecular docking result is only a software simulation, the binding ability of protein to drugs should still confirmed by activity identification. Nevertheless, our study shows that the software simulation had some reference value, but there still has some discrepancy.

### Evaluation of the stability of BlaR-CTD mutant proteins

In a comparison of wild-type protein and single point mutation (I188K), it has been observed that disulfide bond mutation in I188K/S19C/G24C had a better stability and the activity of protein remained longer (Fig. 3), indicating that the disulfide bond played an important role in the stability of protein [10, 18, 22, 30]. I188K/S19C/G24C was more stable than A138E/R50C/Q147C and S190Y/E183C/I188C at 4 °C, 25 °C and 37 °C (Fig. 3). The disulfide bond of mutant protein I188K/S19C/G24C was formed in a flexible region, which is between the last AA of β2 and before the flexible region of α1 of the protein. However, the disulfide bonds of mutant proteins A138E/R50C/Q147C and S190Y/E183C/I188C formed in a more rigid region, which was behind the flexible region of α3 and α7, and between β4 and β5, respectively (Table 3). The different positions of disulfide bond may cause a different effect on the protein stability. The disulfide bonds formed in the flexible region of the protein would prevent the production of extra tension and was beneficial to the stability of the protein [2].

Moreover, when a single point mutation was combined with the introduction of disulfide bonds, it produced the superposition effect in the enhancement of protein affinity (Tables 4 and 5) and stability (Fig. 3). In mutant protein I188K/S19C/G24C, the mutational site I188K was located on the fragment of β5, which was close to the active pocket of BlaR-CTD protein. It might affect the affinity of protein, made the mutation bind to the various β-lactams and enhances the binding affinity of β-lactams. S19C/G24C, located between α1 helix and the flexible region in front of the β2 fragment, were far away from the active site of BlaR-CTD protein and would not change the structure of the active pocket, thus not affect the affinity of the protein.

## Conclusions

To our best knowledge, our study is a new attempt on the structural modification of protein which may be used in the receptor assay for detecting β-lactam antibiotics residues. In this study, the structure of BlaR-CTD protein from *Bacillus licheniformis* ATCC14580 was modified by site-directed mutagenesis based on the combination use of computer simulations, including homologous modeling, molecular docking, disulfide bond inserting and salt bridge building as well as prediction and evaluation of mutation site by software. Mutant protein I188K/S19C/G24C was screened out from 23 mutant proteins for the best stability and higher affinity to 33 β-lactams. As expected, the study results of I188K/S19C/G24C showed that single point mutation combined with disulfide bond insertions could simultaneously increase the recognition ability and stability of the protein. In subsequent studies, mutant proteins I188K/S19C/G24C can be applied to establish a fast and effective receptor method for the screening of β-lactam antibiotic residues.

## Additional file

Additional file 1: **Table S1.** Amino acid residues within 5 Å in the active pocket. **Table S2.** The mutational sites with score of 1.0 predicted by SIFT. **Table S3.** Scoring results by PolyPhen software. **Table S4.** Determination of free sulfhydryl group in mutant protein. **Table S5.** Acitivity identification of BlaR-CTD wild-type and mutant protein using HRP-AMP. **Table S6.** Binding sites of I188K/S19C/G24C to β-lactam antibiotics. **Figure S1.** Expression of BlaR-CTD protein. **Figure S2.** Standard curve of cysteine based on DTNB. **Figure S3.** The DNA and amino acid sequences of recombinant wildtype BlaR-CTD protein. (DOCX 120 kb)

## Abbreviations

3D: Three-dimensional
AA: Amino acid
BlaR-CTD: The C-terminal domain of BlaR
BSA: Bovine serum albumin
HRP-AMP: Horseradish peroxidase-labeled ampicillin
IPTG: Isopropy-β-d-thiogalactoside
LB: Luria–Bertani
PBP: Penicillin binding protein
PBS: Phosphate-buffered saline
TMB: 3,3′,5,5′-tetramethylbenzidine
