# Supplementary material for: Analysis of the stability and affinity of BlaR-CTD protein to β-lactam antibiotics based on docking and mutagenesis studies
Source: J Biol Eng. 2019 Mar 29;13:27. doi: 10.1186/s13036-019-0157-4 (PMC6441189; doi:10.1186/s13036-019-0157-4)
Supplement: Supplementary file 1 — Table S1. Amino acid residues within 5 Å in the active pocket. Table S2. The mutational sites with score of 1.0 predicted by SIFT. Table S3. Scoring results by PolyPhen software. Table S4. Determination of free sulfhydryl group in mutant protein. Table S5. Acitivity identification of BlaR-CTD wild-type and mutant protein using HRP-AMP. Table S6. Binding sites of I188K/S19C/G24C to β-lactam antibiotics. Figure S1. Expression of BlaR-CTD protein. Figure S2. Standard curve of cysteine based on DTNB. Figure S3. The DNA and amino acid sequences of recombinant wildtype BlaR-CTD protein. (DOCX 120 kb) [file 13036_2019_157_MOESM1_ESM.docx]

**Table S1 Amino acid residues within 5Å in the active pocket**

| Ala52 | Pro53 | Ala54 | **Ser55** | **Thr56** | **Tyr57** | **Lys58** | Val59 |
| --- | --- | --- | --- | --- | --- | --- | --- |
| Phe60 | Ser61 | Ala62 | Leu63 | Leu64 | Glu89 | Trp90 | Gln94 |
| Leu96 | Ser98 | Ala99 | Met100 | Ser101 | Ser102 | **Ser103** | **Ala104** |
| **Thr105** | Trp106 | Tyr107 | Phe108 | Gln109 | Lys110 | Asp112 | Leu120 |
| Tyr123 | Leu124 | Ile127 | Tyr129 | Ala138 | Tyr140 | Trp141 | Leu142 |
| Gln147 | Ile148 | Gln153 | Ser178 | Ile179 | Leu181 | Ser190 | Gly191 |
| **Lys192** | **Thr193** | **Gly194** | Thr195 | Ser196 | Ala204 | Gly205 | Trp206 |
| Phe207 | Val208 | Gly209 | Tyr220 | Ala231 | Gly232 | Ser233 | Ala235 |
| Ala236 |  |  |  |  |  |  |  |

Note: The overstriking indicated the AAs of active site and the underlined represented the AAs involved in hydrogen bond forming.

**Table S2 The mutational sites with score of 1.0 predicted by SIFT**

| A11E | E16V | D18L | S19N | F21Y | S26E | G28S | S34E |
| --- | --- | --- | --- | --- | --- | --- | --- |
| N35K | R36E | K37D | T40E | R44K | K45E | A49E | F51V |
| **A52S** | **A54N** | **V59I** | **F60Y** | S68E | K73P | N74E | Q77Y |
| M78L | T79K | T83E | Q84E | Y87F | K88E | E89S | Q92E |
| F97E | S101E | S102N | A104V | **K110E** | R113E | I115V | E117Y |
| H119E | R121K | R122S | S128H | F134L | P137E | **A138E** | G144S |
| **Q147K** | N155E | M156L | Q169E | S170K | **S178A** | R180Y | E182S |
| E183S | N185E | G186E | R187Y | I188E | **S190Y** | V197E | E201K |
| L202D | H203V | **A204N** | **V208I** | A214E | E215D | F218Y | K228D |
| R229N | **A231T** | S233K | T234K | A239T | D244K | K245D | P250E |

Note: The overstriking mutational sites indicated AAs within 5Å of the active pocket but exclude AAs in the active sites and AAs forming hydrogen bond to drugs.

**Table S3 Scoring results by PolyPhen software**

| Mutational site | Score | Mutational site | Score | Mutational site | | Score |
| --- | --- | --- | --- | --- | --- | --- |
| A52S | 0.102 | A54N | 0.082 | | V59I | 0.048 |
| F60Y | 0.106 | K110E | 0.121 | | **A138E** | 0.038 |
| **Q147K** | 0.001 | S178A | 0.167 | | **S190Y** | 0.024 |
| A204N | 0.104 | V208I | 0.103 | | A231T | 0.138 |

Note: The overstriking mutational sites indicated the 3 mutational sites with the lowest score.

**Table S4 Determination of free sulfhydryl group in mutant protein**

| Mutant protein | Absorbance value | C_(Cysteine)_(mM) |
| --- | --- | --- |
| BlaR-CTD | -0.012728 | -0.005689509 |
| S19C/G24C | -0.0091324 | -0.004082249 |
| R50C/Q147C | -0.0050149 | -0.002241697 |
| S76C/L96C | 0.0028305 | 0.001265254 |
| S135C/S145C | -0.023145 | -0.010345984 |
| E183C/I188C | 0.063893 | 0.028560637 |

**Table S5 Acitivity identification of BlaR-CTD wild-type and mutant protein using HRP-AMP**

| Protein | ND | Ampicillin | Penicillin G | Cefoperazone | Nafcillin |
| --- | --- | --- | --- | --- | --- |
| ND | 0.120 | 0.116 | 0.086 | 0.081 | 0.069 |
| BSA | 0.137 | 0.136 | 0.079 | 0.104 | 0.079 |
| BlaR-CTD | 2.207 | 0.134 | 0.114 | 0.115 | 0.205 |
| A138E | 2.489 | 0.171 | 0.140 | 0.121 | 0.398 |
| Q147K | 2.560 | 0.160 | 0.123 | 0.121 | 0.303 |
| I188K | 1.773 | 0.157 | 0.150 | 0.115 | 0.184 |
| S190Y | 1.265 | 0.130 | 0.105 | 0.111 | 0.149 |
| V197D | 2.319 | 0.364 | 0.160 | 0.229 | 0.321 |
| S19C/G24C | 2.472 | 0.347 | 0.340 | 0.187 | 0.413 |
| R50C/Q147C | 2.510 | 0.159 | 0.108 | 0.108 | 0.255 |
| S76C/L96C | 0.350 | 0.13 | 0.138 | 0.133 | 0.133 |
| S135C/S145C | 0.460 | 0.116 | 0.098 | 0.101 | 0.120 |
| E183C/I188C | 2.018 | 0.197 | 0.124 | 0.137 | 0.396 |

**Table S6 Binding sites of I188K/S19C/G24C to β-lactam antibiotics**

| β-lactams | The AA sites involved in the formation of hydrogen bonds to β-lactams | Score |
| --- | --- | --- |
| Pivampicillin | S55, M100, S103, K192, T193, T195 | 6.86↓ |
| Moxalactam | Y87, S55, K192, T195 | 4.93↑ |
| Cefapirin | K58, M100, S103, K192 | 4.78↓ |
| Cefalotin | Y87, S55, S103, T195 | 5.19↓ |
| Ceftazidime | E89, M100, S101, K192, T195 | 5.03↓ |
| Cloxacillin | S55, S103, T105, T195 | 3.89↓ |
| Carbenicillin | S55, S101, S103 | 4.37↓ |
| Cefoxitin | S55, S102, S103, T195 | 7.84↑ |
| Cefamandole | S55, S103, T105, T195 | 6.65↑ |
| Floxacillin | Y87, T105, G194 | 3.59↓ |
| Cefotaxime | S55, K58, S101, S103, T105, K192, T193 | 3.65↓ |
| Cefepime | E89, S55, S103, T105, T195 | 5.54↓ |
| Cefradine | S55, S103, T105, T195 | 6.59↓ |
| Cefalonium | S101, S103, K192, T193, G232, S233 | 4.72↓ |
| Cefquinome | S55, S103, T195 | 4.91↓ |
| Penicillin G | S55, S103, T105, T195 | 5.54↑ |
| Cefuroxime | S55, S101, S103, T195 | 6.65↑ |
| Dicloxacillin | S55, S103, T105, T195 | 4.71↓ |
| Cefaclor | S55, S103, T105, T195 | 6.31↑ |
| Cephradine | S55, S103, T105, T195 | 6.59↑ |
| Cefazolin | S55, S103, T105, T195 | 7.37↑ |
| Imipenem | S55, Y87, E89, T195, G232, S233 | 4.89↑ |
| Ampicillin | S55, S103, T105, G194, T195 | 4.86↑ |
| Sulbenicillin | Y87, S101, T195 | 3.43↓ |
| Ticarcillin | S55, S103, T105, T195 | 4.52↑ |
| Cefalexin | S55, E89, S103, T105 | 4.06↓ |
| Cefatriaxone | S55, E89, S103, T105 | 6.71↑ |
| Methicillin | K58, S103, K192, T193 | 4.81↑ |
| Piperacillin | S55, S103, T105, T195 | 5.01↑ |
| Azlocillin | S55, S103, T105, T195 | 5.31↑ |
| Cefadroxil | S55, S103, T105, T195 | 5.61↑ |
| Nafcillin | S55, S103 | 6.22↑ |
| Cephalosporin | S55, S103, T105, T195 | 7.31↑ |
| Amoxicillin | Y87, T105, T193 | 3.41↓ |
| Furbenicillin | S55, S103, T195 | 5.50↑ |
| Oxacillin | S55, S103, T105 | 3.14↓ |
| Aztreonam | S55, S103, T105, W141, T193, T195 | 4.53↑ |
| Ceftiofur | E89, S55, S103, T195 | 4.04↑ |
| Cefoperazone | S55, S103, T195 | 4.76↑ |
| Cefminox | S55, S101, S103, T193 | 4.55↑ |

Note: The arrow "↑" indicated higher score than wild-type proteins. The arrow "↓" indicated lower score than wild-type proteins.

**Figure legend**

**Fig. S1 Expression of BlaR-CTD protein.** Lane 1 was the total cellular protein of *E. coli* BL21(DE3)/pET-28a(+)-BlaR-CTD after induction by IPTG; lane 2 was the soluble prtotein of the sonicated bacterial cell lysates; lane 3 was the insoluble proteins of the sonicated bacterial cell lysates. M, protein mass marker.

**Fig. S2 Standard curve of cysteine based on DTNB.** The abscissa is the concentration of cysteine and the ordinate is the absorbance at 412 nm.

**Fig. S3 The DNA and amino acid sequences of recombinant wildtype BlaR-CTD protein.** The recombinant protein included BlaR-CTD protein (amino acid number 1 to 254) and an N-terminal extension peptide containing His tag from pET28a vector (amino acid number -34 to -1).

**Fig.S1**


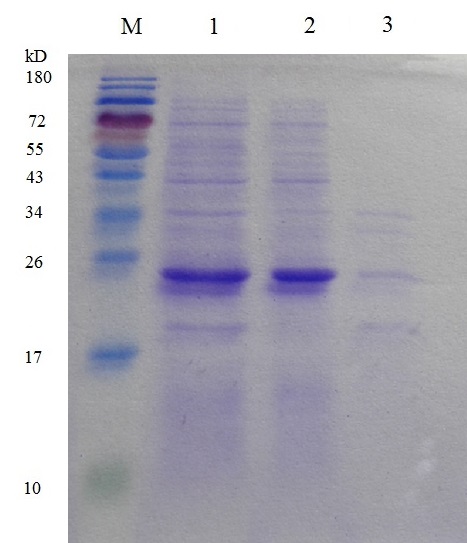


**Fig.S2**

**Fig.S3**

**
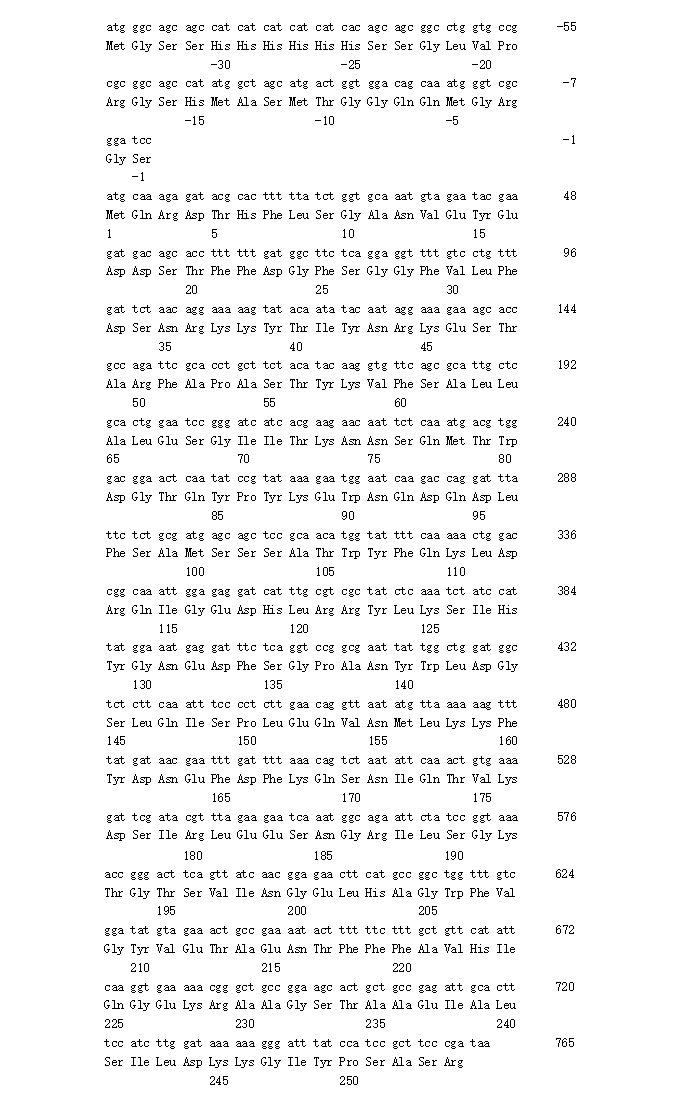
**
